# mHealth Applications to Monitor Lifestyle Behaviors and Circadian Rhythm in Clinical Settings: Current Perspective and Future Directions

**DOI:** 10.3389/fpubh.2022.862065

**Published:** 2022-07-18

**Authors:** Iolanda Rosa, Marlene Lages, Carlos Grilo, Renata Barros, Maria P. Guarino

**Affiliations:** ^1^School of Technology and Management, Polytechnic of Leiria, Leiria, Portugal; ^2^ciTechCare - Center for Innovative Care and Health Technology, Polytechnic of Leiria, Leiria, Portugal; ^3^Faculty of Nutrition and Food Sciences, University of Porto, Porto, Portugal; ^4^EPIUnit, Instituto de Saúde Publica, Universidade do Porto, Porto, Portugal; ^5^Laboratório Para a Investigação Integrativa e Translacional em Saúde Populacional (ITR), Universidade do Porto, Porto, Portugal; ^6^Computer Science and Communication Research Center, Polytechnic of Leiria, Leiria, Portugal; ^7^School of Health Sciences, Polytechnic of Leiria, Leiria, Portugal

**Keywords:** mHealth, chronic care management, metabolic diseases, circadian rhythms, nutrition, sleep, physical exercise, usability

## Abstract

Metabolic diseases are a global rising health burden, mainly due to the deleterious interaction of current lifestyles with the underlying biology of these diseases. Daily habits and behaviors, such as diet, sleep, and physical exercise impact the whole-body circadian system through the synchronization of the peripheral body clocks that contribute to metabolic homeostasis. The disruption of this system may promote the development of metabolic diseases, including obesity and diabetes, emphasizing the importance of assessing and monitoring variables that affect circadian rhythms. Advances in technology are generating innovative resources and tools for health care management and patient monitoring, particularly important for chronic conditions. The use of mobile health technologies, known as mHealth, is increasing and these approaches are contributing to aiding both patients and healthcare professionals in disease management and education. The mHealth solutions allow continuous monitoring of patients, sharing relevant information and data with physicians and other healthcare professionals and accessing education resources to support informed decisions. Thus, if properly used, these tools empower patients and help them to adopt healthier lifestyles. This article aims to give an overview of the influence of circadian rhythms disruption and lifestyle habits in the progression of metabolic diseases while also reviewing some of the mobile applications available to monitor lifestyle behaviors and individual chronobiology. Herein is also described the design and development of the NutriClock system, an mHealth solution developed by our team to monitor these variables.

## Introduction

The prevalence of metabolic diseases, particularly obesity and type 2 diabetes mellitus (T2DM), continues to rise globally. Obesity has grown to epidemic proportions, with over 4 million annual deaths attributed to overweight in 2017 (1). This condition is also an established risk factor for T2DM, with around 80% of people with T2DM being obese. In the next 25 years, it is expected that more than 600 million people will be diagnosed with T2DM, and around the same number of people will develop impaired glucose tolerance, a state that frequently precedes T2DM (2).

These statistical predictions, in combination with the global epidemic of the novel coronavirus (COVID-19), heightened an additional need for valid and reliable technology in health care. This includes the development and improvement of digital platforms dedicated to assisting professionals in providing health care to patients whenever face-to-face interactions are not viable (3). The proliferation of mobile health apps (mHealth) has fostered their use in delivering health-related behavior change interventions (4). These apps are now commonly used to self-monitor lifestyle behaviors and some even allow sharing of information with healthcare professionals (3).

Several mHealth apps have been developed to assist patients in managing chronic diseases, such as metabolic diseases. Through education, diet, activity tracking, and personalized health advice, these apps have the potential to guide behavior change. Dietary changes combined with healthy lifestyle adjustments should be the first approach since they have been identified as one of the most effective interventions for preventing, managing, and reversing chronic conditions. mHealth apps designed to support these actions may have a central role in health promotion and care (4).

The relevance of circadian rhythms in the regulation of energy metabolism, as well as the role of circadian disruption in poor health outcomes, is well-documented in the literature, both in clinical and non-clinical experimental settings (5). These rhythms are driven by an endogenous mechanism and enable our body to achieve optimal functional performance. Circadian rhythm disruption is associated with an increased risk of developing metabolic diseases (6, 7). Patients and healthcare professionals would benefit from access to standardized and clinically validated technology to monitor disrupted circadian rhythms and implement personalized nutritional approaches. Due to the widespread use of smartphone devices across the world, mHealth applications already represent a useful resource in clinical settings.

The present article aims to provide an overview of the current clinical evidence regarding the impact of circadian rhythms on the development of metabolic diseases and summarize the mobile applications available to monitor lifestyle behaviors and individual chronobiology, highlighting the innovative aspects brought by the NutriClock mHealth solution.

## Circadian Rhythm, Metabolic Homeostasis, And Lifestyle Behaviors

Metabolic homeostasis is a critical component that regulates energy metabolism. In recent years, several studies enlightened the relationship between human physiology, the circadian rhythm and a cluster of metabolic diseases, including T2DM and obesity (8, 9).

The circadian rhythm, also known as the biological clock, refers to the physiological, molecular and behavioral changes that occur during a cycle of approximately 24 h. In humans, these rhythms are synchronized by a hierarchical system: the central clock in the suprachiasmatic nucleus (SCN) of the hypothalamus and the peripheral clocks, located in several organs and tissues throughout the body (10). While the SCN is mainly synchronized by the light/dark cycles, the peripheral clocks react to other stimuli, including the feeding/fasting state, nutrients, sleep-wake cycles, and physical activity (11, 12).

Daily eating patterns are affected by several factors, including, hunger and satiety, social habits and food availability. Nonetheless, when eating occurs at a consistent and expected schedule, the circadian clock system initiates nutrient-sensing pathways to uphold nutrient homeostasis (13). When eating schedules are altered to occur at random times, these same nutrient-responsive pathways give feedback to the circadian clocks to adjust their phase shift. This disruption acutely impacts glycemic control and insulin sensitivity, increasing the risk of developing T2DM (14). Besides insulin sensitivity, the effects of the circadian system are seen in numerous metabolic and hormonal rhythms, including cortisol and melatonin. These two anti-phasic hormones are involved in the signaling between the master clock and the other peripheral oscillators (15, 16) and can be used to evaluate circadian rhythms, though their measurement can be affected by external factors (17). However, presently, the most straightforward method to study and analyse circadian-derived behavior in humans consists of measuring physical activity.

These findings highlight the importance of measuring variables related to circadian behavior, such as eating schedules, physical activity and circadian biomarkers, to potentiate the effects of current nutritional and medical approaches in the management and prevention of chronic metabolic diseases.

## Mobile Solutions To Monitor Outpatients' Circadian Rhythms And Health

The *my Circadian Clock* (mCC) application was developed as part of a chrononutrition-related study to assess the influence that the timing of caloric intake has on metabolic homeostasis and subsequent prognosis of pathology. The 156 participants were asked to record all the meals they ingested. They received app notifications once or twice a day, at random times, to check if they had eaten in the last 30 min and forgotten to record the event. The false-negative rate was estimated to be 10.34%. The results showed that more than half of the participants had an erratic eating pattern. According to the data inserted in the mCC application, the participants spent most of the day consuming calories, with only a brief period of fasting. Furthermore, a higher caloric intake was observed in the afternoon and evening, which could have several consequences, including a negative impact on sleep quality (18).

More recently, Wilkinson and colleagues (19) also used the mCC app in their study to assess the impact of 10-h time-restricted eating (TRE) on 19 patients with Metabolic Syndrome (MetS). The mCC app was used during baseline and the intervention to register food/beverage intake and sleep. The adherence to logging information in the app was 94.30 ± 7.25% during the 2-week baseline, and 85.61 ± 12.39% during the 12-week intervention (19). Caloric intake was estimated based on photos and/or annotations since the mCC app is not designed to enter the exact food portions. The results of this study showed a general reduction in body weight, waist circumference, blood pressure, glycated hemoglobin and an improvement in lipid profile and sleep.

Sakane and colleagues (20) assessed the impact of a chrononutrition-based mobile application on weight changes. The study included 1 835 participants that used the app “Reborn Magic” for 4 weeks to behavior self-monitoring. Aside from weight changes, other outcomes assessed included waking time, breakfast, lunch, and dinner timing, weekend and holiday bedtime, and physical condition score. According to the findings, age was negatively correlated with waking time and dinner timing. Inappropriate meal timing was found in 32.9% of dinners, 34.2% of lunches and 61% of breakfasts. After the intervention, the physical condition scores increased significantly from 6.6 to 8.2 points out of 10. Significant weight loss in participants with overweight and obesity was observed, while significant weight gain was observed in lean participants. Normal-weight participants experienced no weight changes. The authors concluded that chrononutrition-based apps may be effective for weight loss. However, additional research, including randomized controlled trials (RCT), is needed (20), particularly to clarify the individual effects of TRE on metabolic diseases independent of caloric restriction.

Tahara and colleagues (21) conducted a study on Japanese people to assess if changes in chronotype, due to the recent pandemic confinement, were associated with body weight changes. They analyzed data collected from 30,275 Asken mobile app users via an online survey. The results revealed an association between changes in sleep stages and body weight. During a mild lockdown, participants who reported advanced sleep phases on both work and non-workdays lost weight and those who slept later gained weight. Despite these findings, the authors highlighted that the study has methodological limitations, so more research is needed to validate these findings.

Swiatkiewicz and colleagues (22) coordinated the TREMNIOS study, a pilot clinical trial conducted in Polish adults with MetS and an eating period >14 h per day. The study also uses the mCC app to assess if TRE could help patients regain rhythmic daily behavior and improve cardiometabolic outcomes. Participants are expected to complete a 10-h TRE intervention for 24 weeks, and changes in the eating window, body weight, and biomarkers will be assessed. Metabolic, neuroendocrine, inflammatory and antioxidant biomarkers, body weight and composition, blood pressure, heart rate, sleep and activity, personal sense of wellness and dietary timing will be evaluated at the baseline and after intervention with compliance with TRE assessed using the app. The study is estimated to be complete in January 2023 and it is expected that these results can set the foundation for a large-scale RCT to determine the efficacy and sustainability of TRE in reducing long-term cardiometabolic risk in patients with MetS.

## NutriClock: Development of a New App and Web Platform to Monitor Circadian Behaviors

The NutriClock (National Trademark Registration no. 664951) mHealth platform is a deliverable of the NutriClock study, whose primary goal is to serve as a data collection, monitoring, and analysis support tool. The NutriClock app collects data related to lifestyle behaviors including sleep, physical activity, dietary patterns, and circadian rhythm biomarkers. The multidisciplinary team behind the design and development of this platform included computer engineers, nutritionists, physiologists, and psychologists.

The platform was built using agile methodologies, which enable iterative, incremental, and adaptable system implementation in response to changing requirements, as well as continuous feedback on potential improvements.

**Figure 1:**
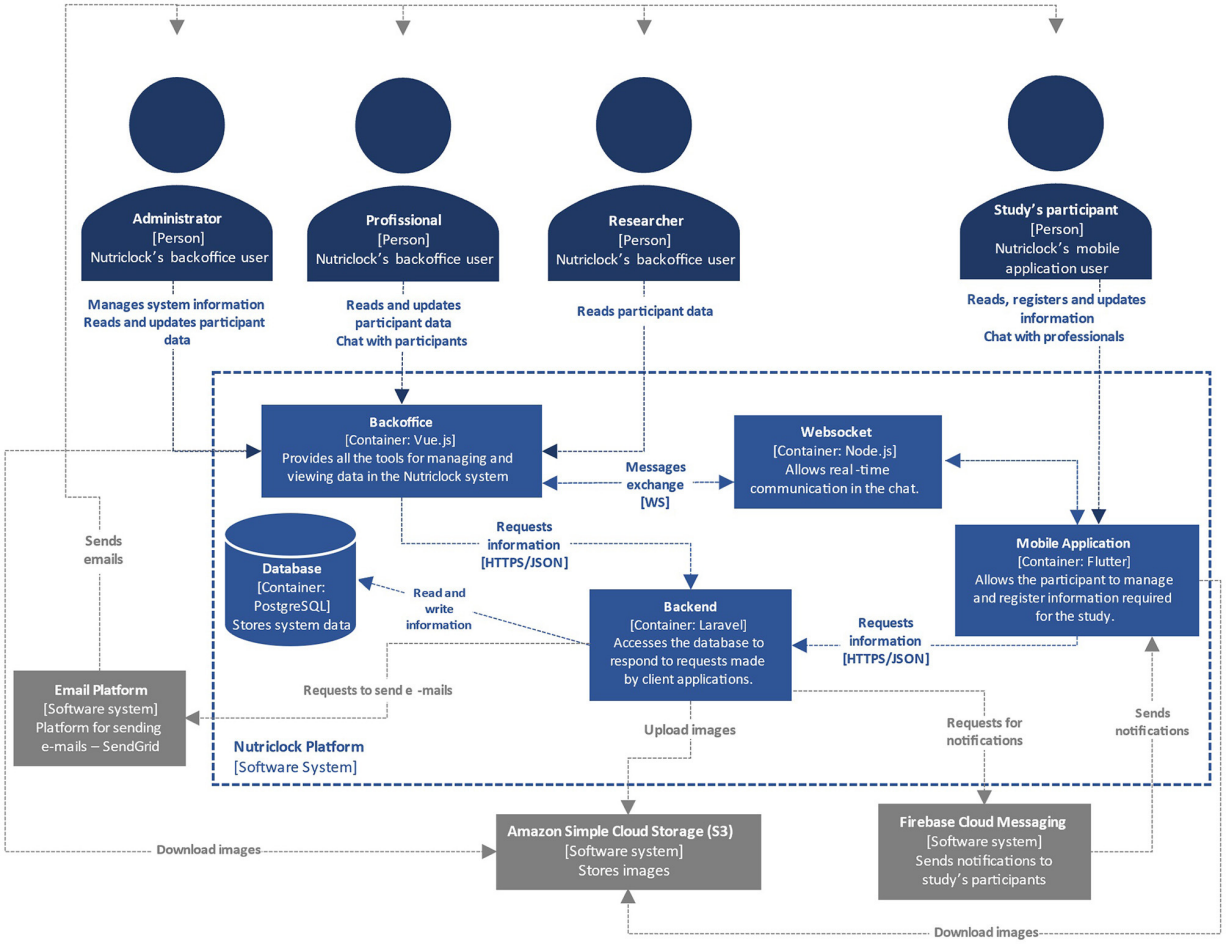
NutriClock system architecture diagram – level 2 of the C4 model.

Figure 1 depicts the NutriClock system's high-level architecture diagram using Level 2 of the C4 model (23). The C4 model is a set of diagrams used to represent software architecture at four different levels of abstraction: Context (Level 1), Container (Level 2), Component (Level 3), and Code (Level 4) diagrams. Level 2 depicts the high-level shape of the software architecture, the major technology choices, and how the containers communicate with one another. Figure 1 shows that the platform is divided into four modules: the backend, which is the system's foundation; the backoffice, used by administrators, healthcare professionals, and researchers; the mobile application, used by study participants; and a WebSocket service that allows real-time communication via chat. Each module serves a specific purpose but for the system to work, modules must communicate with one another and with external services for sending notifications, emails, and storing images. Screenshots of the mobile app and backoffice are available in the Supplementary Material.

The backend module is a REST API that writes and reads data from the database to provide the information requested by client applications. The available resources are documented at http://nutriclock.herokuapp.com/api/documentation, and are protected by authentication mechanisms. Furthermore, middlewares are used to parse and filter client requests to increase security. To store images, send emails, and manage user notifications, the backend communicates with external services.

### The NutriClock Backoffice

The backoffice is a single-page web application designed to provide a platform for administrators, healthcare professionals, and researchers to manage and analyse data from participants. To protect and limit access to participants' data, each user role has different management and access permissions. As an example, healthcare professionals can only access the data of the participants registered in their healthcare institution.

The following are some of the main features available in the backoffice:

1 Authentication and user profile management;
2 Management of the participants' data (available for all users with differentiated privileges), including:
3 Create and manage personalized meal plans by a registered nutritionist/dietitian;
4 Monitor and analyse the data from the sleep, food, and physical activity diaries;
5 Manage the list of participants (block, unblock, or remove participants);
6 Communicate with the participants via chat (bidirectional exchange). This can be a motivational feature for the participants;
7 Select the participant to the control or experimental arm of the study (for example, it automatically opens the meal plan option);
8 Check if the participant has entered the information necessary for the study through a color system (green, yellow and red). The system will automatically send reminders if there is information missing;
9 Create, update, or delete information, including the professional categories, institutions, pathologies, food database, physical activity database, biomarkers, and terms of acceptance (available for the administrators only). These features were included to help the administrators manage the information available in the app and will be used strictly following the Good Clinical Practice.
10 View the NutriClock mobile app ratings (available for the administrators only).

### The NutriClock Mobile Application

The mobile application provides an interface for participants to enter the data and communicate with healthcare professionals via NutriClock's chat. Besides, the application will notify them when data is missing in their meal, sleep, or physical activity diaries, and when saliva and urine samples should be collected. The following are some of the features available:

1 Create a new account and authentication related functionalities;
2 Sleep diary to assess quantity and quality of sleep;
3 Physical activity diary (there is an option to select physical activity or physical exercise);
4 Food diary (enter the food item or meal, quantity, nutritional information and pictures);
5 Chat where the participant can message the healthcare professional;
6 Access to contacts, meal plans, reports and biomarker information (schedule of biological samples collections to determine cortisol and melatonin);
7 Manage the notifications settings (turn on/off);
8 Option to delete the account and data according to the right to be forgotten;
9 Rating of the NutriClock application using a 5-points Likert scale.

## Preliminary Usability Tests of the Nutriclock System

The design and development of mobile applications with an easy-to-use interface is a crucial step for their successful adoption in long term (24). Usability tests represent an assessment method to measure how well-users can interact with the system and how easy and intuitive it is to execute the functions of the mobile application (25). Conducting usability tests is critical and preferably these should cover all or the majority of possible situations, however, this represents a major challenge. So, usability tests may focus only on some features of the mobile application (26). According to Zhang and Adipat (26), usability testing can be performed with laboratory experiments in which participants are required to complete specific tasks using the mobile application in a controlled setting.

Testing the usability of mobile applications is constrained by several factors, including small screen size and limited technological performance compared to other devices. Apps' ease of use and acceptance is linked with users' perception of time consumed when performing tasks, easy-to-learn functions, and user-friendly features (27), characteristics that were assessed in the NutriClock pilot-usability testing. It is also reported in the literature that mHealth apps may be more effective in creating meaningful behavior change interventions when designed to support end-user values and needs, assessed by persuasive system design principles (28). These tools were not used in the NutriClock system development.

In the case of the backoffice application, the development process focused mainly on functionality. However, usability tests conducted on this web application are also important to identify usability related issues that were improved, for example, providing feedback regarding actions taken by users (29, 30).

The NutriClock system was tested using manual, automated, and usability tests. Manual tests were primarily used to validate features and identify changes and improvements to be made to meet the platform requirements. Automated tests helped in the discovery of bugs that were missed during manual testing. Usability tests were useful to improve the user interface and to solve usability problems identified by test users, for example, knowing how to access some sections of the mobile application.

### Usability Tests Methods

To participate in the usability tests, people had to be over 18 years old, provide their informed consent and not have any prior contact with the NutriClock system (to allow the assessment of the system's usability and intuitiveness by observing the users' behavior during the initial interactions). No exclusion criteria were applied.

To conduct the usability tests, the researchers created three scripts which included two scripts to test the mobile application with a patient account covering different sets of features of the NutriClock app (seven tasks in each script) and one script with 6 tasks to test the backoffice using administrator and healthcare professional accounts. The three scripts with the complete tasks are detailed in the Supplementary Material. To perform the backoffice test, a computer with internet access and an installed browser application were used. The mobile application was tested using either a Huawei tablet, model MediaPad M5 Lite or a Xiaomi Pocophone smartphone, model F1.

Statistical analysis was performed using SPSS 27.0 to assess the differences in execution time between participants with equal or <30 years (subgroup A) and participants with more than 30 years (subgroup B) for the seven tasks of test no. 1, 7 tasks of test no. 2 and 6 tasks of test no. 3. The age cut-off point for the subgroups was selected according to the distribution of age ranges of the total sample. Statistical significance was set at *p*-value < 0.05.

### Preliminary Results

Following the Zhang and Adipat (26) methodology, 13 participants (nine women and four men) with a mean age of 35.62 ± 12.16 years (age range: 21–58 years) performed the usability tests. The tests had to be conducted in person, however, due to COVID-19 restrictions at the time, it was not possible to recruit a larger sample. Nine (69.2%) participants completed all three tests, two (15.4%) completed two tests and two (15.4%) only performed one, the criteria being their time availability.

### Usability Test No.1 – Test NutriClock App as Patient

**Figure 2:**
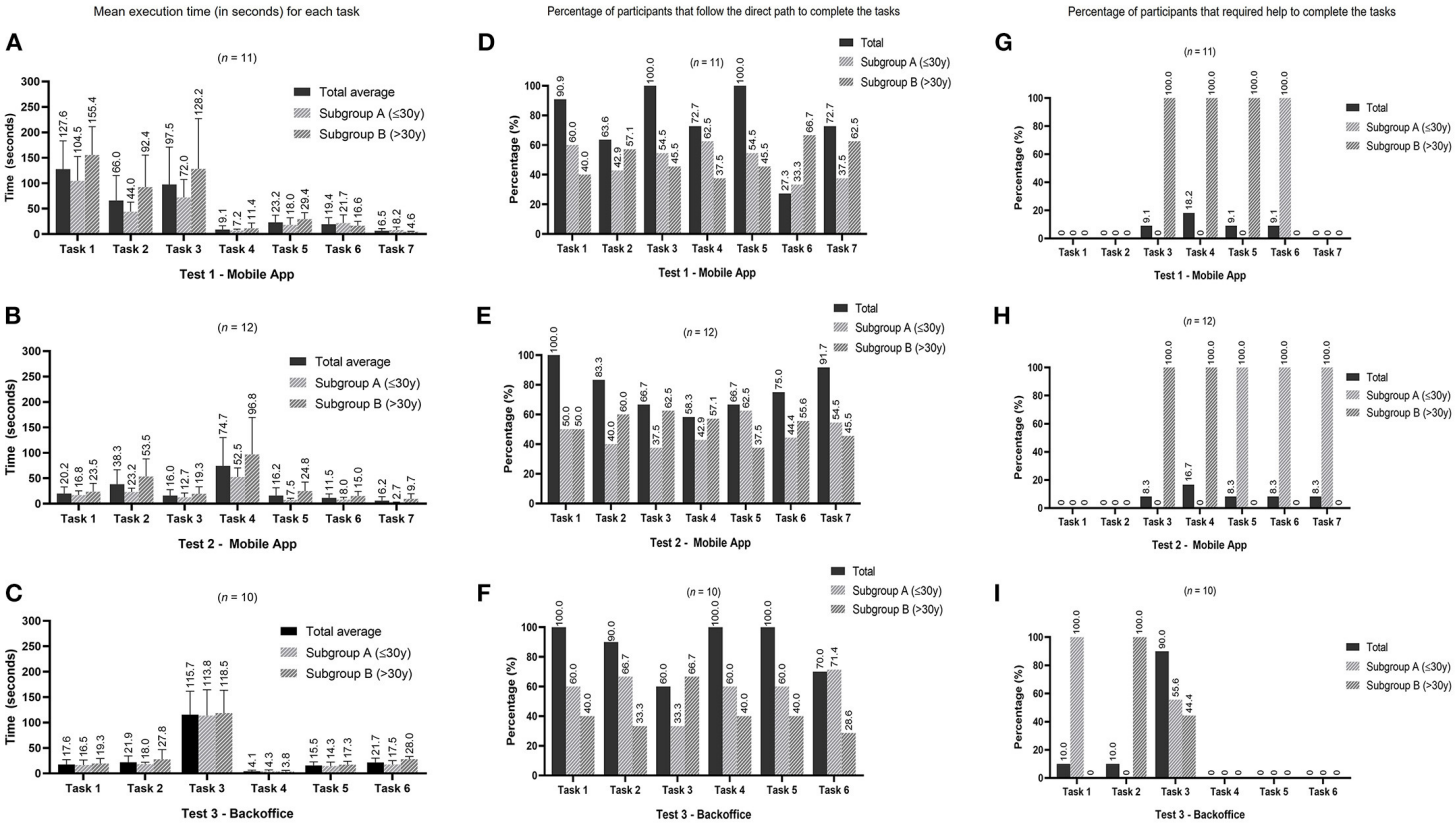
Graphics display the results of the three different usability tests by tasks. Graphics **(A–C)** show the mean execution time (values placed above the standard deviation bar). Graphics **(D–F)** represent the percentage of participants that immediately follow the direct path to complete the tasks defined in the three tests. The percentages of subgroups **(A,B)** are relative to the number of participants who followed the expected path. Graphics **(G–I)** represent the percentage of participants that required assistance from the researcher to complete the tasks in the three tests. The percentages for subgroups **(A,B)** are relative to the number of participants who needed help.

Script no.1 for the NutriClock app, was conducted on 11 people and took, on average, 6 min per participant to complete the seven tasks. Participants took longer to complete tasks 1 and 3 (Figure 2A), which were creating a new account and inserting an entry in the food diary, respectively. These were indeed the most time-consuming tasks and since 90.9% (task 1) and 100% (task 3) of the participants followed the expected path (defined as the quickest and most direct way to perform the function) (Figure 2D) and only 9.1% needed help from the researchers to perform task 3 (Figure 2G) it was considered that these features were simple to understand. However, in task 6, in which the participants were asked to view the biomarkers section, 72.7% of them did not follow the expected path (Figure 2D) because they needed to explore the app to find this section since this is more specific for this app and not a general feature. Interestingly, in this task subgroup A had the worst performance in all three indicators compared to subgroup B which seems to indicate that they had to explore more the application to find this menu. Regarding task 7, there were two paths to logout of the application, and a smaller percentage of participants in subgroup A seems to have chosen the direct path, which led them to take, on average, longer to complete this task compared to subgroup B. Overall, in this test, the expected path for executing the tasks was followed by 75% of the participants and only 7% needed assistance to complete a task.

In this test, there was only a statistical difference in median execution time for task 2 (add an entry in the sleep diary) for subgroups A (38 s) and B (80 s) (Mann-Whitney U = 4.0, n_A_ = 6, n_B_ = 5, *p* = 0.045 two-tailed). Although execution time might be an indicator of the difficulties the participants experienced, in this task, participants could take longer because they had to add more information. For example, a participant who answered that they woke up during the night had to fill in more information than a participant who answered no to this first question.

### Usability Test No.2 – Test NutriClock App as Administrator

This test was performed by 12 people and took, on average, 3 min per participant to complete the seven tasks. Participants took longer to add a new entry in the physical activity diary (task 4) (Figure 2B). This was also the task with the lower percentage of participants (58.3%) following the expected path (Figure 2E) and the higher percentage of participants (16.7%) requiring help to execute it (Figure 2H) which can indicate that the usability of this feature may be improved. In general, the expected path was followed, on average, by 77% of participants, with 7% of participants needing help to complete a task. In this test, subgroup A had a lower percentage of participants following the expected path in tasks 2, 3, 4, and 6, even though they took less time to complete. This can be an indicator that the mobile application is quick to explore but not always intuitive to some users.

A statistically significant difference was found in the median execution times for task 2 (edit information entered in the food diary): subgroup A = 24 s and subgroup B = 48 s (Mann-Whitney U = 5.0, n_A_ = 6, n_B_ = 6, *p* = 0.037 two-tailed). This difference was due to a participant from subgroup B that took 120 s to complete this task, while all the other participants took <55 s. There were also significant differences in median execution times for task 5 (consult notifications settings): subgroup A = 6 s and subgroup B = 22.5 s (Mann-Whitney U = 4.0, n_A_ = 6, n_B_= 6, *p* = 0.024 two-tailed). In this task, the participants that took more than 15 s to complete it were from subgroup B which may explain the difference found in the median execution times. The last statically significant difference was found on median execution times for task 7 (logout application): subgroup A = 2.5 s and subgroup B = 6 s (Mann-Whitney U = 0.5, n_A_ = 6, n_B_ = 6, *p* = 0.004 two-tailed). All of the participants took <10 s to complete this task, except one participant in subgroup B that took 30 s.

### Usability Test No.3 – Testing the Backoffice Using Administrator and Healthcare Professional Accounts

Regarding the backoffice test, 10 participants completed this script, taking, on average 3 min to complete the six tasks. Task 3 (adding a food item to a meal plan) was the one that participants had more difficulty completing. This was the one with a longer execution time (Figure 2C), a lower percentage of participants (60.0%) following the expected path (Figure 2F) and a higher percentage of participants (90.0%) that needed help to complete it (Figure 2I). The results of this test highlighted the need to improve this function and make it more user-friendly.

In general, the expected path for executing a task was followed, on average, by 87 and 18% of participants needed assistance.

No significant differences were found in the execution time between subgroups A and B.

### Study Limitations

Some limitations were identified that should be considered in future studies, the main one being the small sample size. Also, the usability testing in this controlled setting may not represent how the end-user will interact with the mobile application in their daily routines. The participants were not all representative of the real end-users (patients with metabolic diseases) and the age range of the participants included should have been broader to represent all the potential end-users of the NutriClock application. This will be taken into consideration in the next phase of usability tests as well as in the tests to evaluate the perceived quality of the system. In future work, and after improving the issues identified, the app usability will be re-tested based on a validated model and in real-end users. Also, future tests will be performed in the field to assess how people use the app in a real and not controlled environment.

## Future Perspectives of mHealth Solutions to Assist Lifestyle Interventions

The potential of mHealth applications to improve access to healthcare resources and real-time monitoring is already recognized (31–33). Medical health practice supported by mobile devices continues to scale up, and current literature suggests that higher levels of engagement of both patients and healthcare providers are often associated with better health outcomes (34–36). Also, these tools could help healthcare professionals motivate patients in remote settings to adopt healthier lifestyles, manage chronic diseases and reduce complications (37–39).

However, these solutions may also present some issues, particularly concerning data privacy and the protection of sensitive information shared by patients (40). Another challenge faced is the digital gap experienced by some patients and the intellectual capabilities of users which could lead to high attrition rates (41).

To circumvent some of these challenges, when designing a mobile application to address behavior change, it is important to have a comprehensive concept, essential to achieve an ongoing impact (42, 43). McClung and colleagues proposed an approach based on a health-centered design as a solution that would benefit the future of mHealth tools (3). This highlights the importance of starting the development process with a context analysis to identify the primary functions required for the solution, which must meet the needs of all stakeholders involved. Furthermore, a user-centered design is required to create an engaging application (3). The personalization based on user preferences is also relevant for the success of mHealth solutions. This can encompass personalization considering the information on medical factors and personal preferences; disease-specific education tips; the ability to track progress, which includes viewing previous logs and uploading photos; reminders and reinforcement based on user feedback with motivational messages, are some of the characteristics of a successful and engaging mHealth app, according to Joshi and colleagues (4). Also, Salari and colleagues (44) revealed that among 23 minimum set of features for diabetes mobiles apps, mealtime tagging, food database, diet management, educational materials, healthy coping, reminders, target range setting, trend chart view, and numerical indicators view are among the features deemed important by experts. To fulfill all these requirements, interdisciplinary and collaborative work (42) between healthcare professionals, patients, and software development teams is essential throughout the product development process (3).

In the future, evidence-based mHealth solutions may be integrated into traditional clinical treatment approaches to improve health results and access to primary care prevention. This could be particularly important in the context of chronic diseases, such as metabolic diseases since they frequently need to be managed from their onset with few options for a full recovery and these solutions could help patients achieve better results (45, 46). Patients can benefit from mHealth solutions that allow them to self-monitor, stay in control, and be better informed about their health by recording and analyzing information.

This perspective is aligned with the future of the NutriClock app and backoffice, as it is expected that this system can be implemented in clinical settings to help healthcare professionals monitor lifestyle behaviors and individual circadian rhythms and empower patients by giving them an active role in their healthcare monitoring and management process. Besides, this system will also help researchers in the field design clinical studies on chrononutrition and chronobiology, with larger samples since participants can be monitored remotely.

The evidence arising from the use of mHealth solutions appears promising. However, the current published evidence of improvements in patients' health outcomes using these tools is still restricted to a limited number of clinical situations (47). More research should be conducted to investigate the feasibility of these solutions, particularly in the treatment of metabolic diseases, so that the use of these tools can become part of the therapeutic approaches used in clinical settings.

The results of chrononutrition studies are encouraging but more clinical studies with larger samples and longer periods of intervention are necessary before translating the current knowledge to clinical practice. So tools that support these interventions and studies, such as the NutriClock system, are relevant. Currently, there are still improvements to implement in the NutriClock system, including adding reference pictures for the portions, according to the meal or food item entered by the user in the food diary. Another feature that could be important to add would be a time setter and reminders to show the optimum eating window according to the individual chronotype to attempt to control erratic eating patterns. To circumvent the shortcomings of the currently available mHealth applications, especially, the lower engagement of patients, we added the chat function to have a fast direct contact point with the healthcare professionals. Motivational strategies to integrate into the NutriClock app, to lower the dropout rates and increase patients' engagement with this technology, are also being studied. The next steps will include a usability test to assess these new features, followed by a feasibility and acceptability study before starting the clinical validation of the NutriClock system.

## Conclusion

Lifestyle interventions to treat and prevent chronic metabolic diseases need to consider the daily circadian rhythms since aligning those behaviors with the individual biological rhythm potentiates the health benefits. The use of mHealth is increasing and these solutions represent an interesting opportunity to monitor and measure health variables and lifestyle habits in normal living conditions, helping patients and healthcare professionals in disease management. The NutriClock mHealth system collects data related to lifestyle behaviors and will serve as a basis to elaborate meal plans tailored to the individual circadian characteristics, making this an innovative and differentiating solution. In the future, it is expected that this platform becomes a clinically validated therapeutic tool used to integrate individual biological rhythms in the treatment and prevention of metabolic diseases supporting interventions directed to lifestyle changes.

## Data Availability Statement

The raw data supporting the conclusions of this article will be made available by the authors, without undue reservation.

## Ethics Statement

Ethical review and approval was not required for the study on human participants in accordance with the local legislation and institutional requirements. The patients/participants provided their written informed consent to participate in this study.

## Author Contributions

ML, MG, and RB led the design of the study with the contributions of IR and CG. ML and IR drafted the manuscript, with the contributions of MG and CG. All authors contributed to the manuscript, revised it critically for intellectual content and approved the final manuscript submitted.

## Funding

This work was supported by Portuguese National Funds provided by Fundação para a Ciência e Tecnologia (UIDB/05704/2020 and UIDP/05704/2020). ML was supported by a PhD Scholarship from Fundação para a Ciência e Tecnologia (2021.07673.BD).

## Conflict of Interest

The authors declare that the research was conducted in the absence of any commercial or financial relationships that could be construed as a potential conflict of interest. The reviewer RC declared a shared affiliation with the author RB to the handling editor at the time of review.

## Publisher's Note

All claims expressed in this article are solely those of the authors and do not necessarily represent those of their affiliated organizations, or those of the publisher, the editors and the reviewers. Any product that may be evaluated in this article, or claim that may be made by its manufacturer, is not guaranteed or endorsed by the publisher.
