# Supplementary material for: mHealth Applications to Monitor Lifestyle Behaviors and Circadian Rhythm in Clinical Settings: Current Perspective and Future Directions
Source: Front Public Health. 2022 Jul 18;10:862065. doi: 10.3389/fpubh.2022.862065 (PMC9339674; doi:10.3389/fpubh.2022.862065)
Supplement: Supplementary file 1 [file Data_Sheet_1.pdf]

Supplementary Table 1. Usability test script no.1 for the mobile application “NutriClock"

<table>
<tr><td colspan="4">Job / Study area:</td><td colspan="3"></td></tr>
<tr><td>Sex:</td><td></td><td>Age:</td><td></td><td>Smartphone model:</td><td colspan="2"></td></tr>
<tr><td colspan="7">Task #1
Create a new patient account</td></tr>
<tr><td colspan="5">Execution time
How many seconds did the user take to complete the task?</td><td colspan="2"></td></tr>
<tr><td colspan="5">Expected path
Did the user follow the expected path?</td><td>Yes</td><td>No</td></tr>
<tr><td colspan="7">Expected path description
On the login screen select the "New Account" option – Fill out the required fields – Click "Register" and accept the terms and conditions</td></tr>
<tr><td colspan="5">Errors and assistance
Did the user need help?</td><td>Yes</td><td>No</td></tr>
<tr><td colspan="7">The user is lost
How many errors occurred and how did the user expect the application to behave?</td></tr>
<tr><td colspan="7">Task #2
Add a new entry in the sleep diary</td></tr>
<tr><td colspan="5">Execution time
How many seconds did the user take to complete the task?</td><td colspan="2"></td></tr>
<tr><td colspan="5">Expected path
Did the user follow the expected path?</td><td>Yes</td><td>No</td></tr>
<tr><td colspan="7">Expected path description
In the bottom bar select the "Sleep" option – On the screen select "Sleep Diary" – Select the date – Fill in the required fields – Click "Confirm"</td></tr>
</table>

**Errors and assistance**<br>Did the user need help?	Yes	No
**The user is lost**<br>How many errors occurred and how did the user expect the application to behave?		
**Task #3**<br>**Add a new entry in the food diary**		
**Execution time**<br>How many seconds did the user take to complete the task?		
**Expected path**<br>Did the user follow the expected path?	Yes	No
Expected path description<br>*In the bottom bar select the "Diary" option – Select the "+" option – Select the meal, the date and time – Fill in the required fields – Click on "Confirm"*		
**Errors and assistance**<br>Did the user need help?	Yes	No
**The user is lost**<br>How many errors occurred and how did the user expect the application to behave?		
**Task #4**<br>**Delete an entry for the food diary**		
**Execution time**<br>How many seconds does it take to complete the task?		
**Expected path**<br>Did the user follow the expected path?	Yes	No
Expected path description<br>*In the bottom bar, select the "Diary" option. Click to see the details in the meal list. Click the "Delete" option. On confirmation, click "Delete"*		

**Errors and assistance**<br>Did the user need help?	Yes	No
**The user is lost**<br>How many errors occurred and how did the user expect the application to behave?		
**Task #5**<br>**Send a chat message to a healthcare professional**		
**Execution time**<br>How many seconds did the user take to complete the task?		
**Expected path**<br>Did the user follow the expected path?	Yes	No
Expected path description<br>*In the bottom bar, select the "Chat" option. Select a user from the list. Write a message. Click send*		
**Errors and assistance**<br>Did the user need help?	Yes	No
**The user is lost**<br>How many errors occurred and how did the user expect the application to behave?		
**Task #6**<br>**View the biomarkers information**		
**Execution time**<br>How many seconds did the user take to complete the task?		
**Expected path**<br>Did the user follow the expected path?	Yes	No
Expected path description<br>*In the side menu select the option "Biomarkers"*		
**Errors and assistance**	Yes	No

Did the user need help?		
**The user is lost**<br>How many errors occurred and how did the user expect the application to behave?		
**Task #7**<br>**Logout from the application**		
**Execution time**<br>How many seconds did the user take to complete the task?		
**Expected path**<br>Did the user follow the expected path?	Yes	No
Expected path description<br>*In the top bar select the "Logout" option – In the confirmation select the "Yes" option*		
**Errors and assistance**<br>Did the user need help?	Yes	No
**The user is lost**<br>How many errors occurred and how did the user expect the application to behave?		

Supplementary Table 2. Usability test script no. 2 for the mobile application “NutriClock”

<table>
<tr><td colspan="4">Job / Study area:</td><td colspan="3"></td></tr>
<tr><td>Sex:</td><td></td><td>Age:</td><td></td><td colspan="2">Smartphone model:</td><td></td></tr>
<tr><td colspan="7">Task #1
Log into the application with the administrator account indicated by the researcher</td></tr>
<tr><td colspan="5">Execution time
How many seconds did the user take to complete the task?</td><td colspan="2"></td></tr>
<tr><td colspan="5">Expected path
Did the user follow the expected path?</td><td>Yes</td><td>No</td></tr>
<tr><td colspan="7">Expected path description
On the login screen, fill in the fields and click on "Login"</td></tr>
<tr><td colspan="5">Errors and assistance
Did the user need help?</td><td>Yes</td><td>No</td></tr>
<tr><td colspan="7">The user is lost
How many errors occurred and how did the user expect the application to behave?</td></tr>
<tr><td colspan="7">Task #2
View your food diary and update the existing food name.</td></tr>
<tr><td colspan="5">Execution time
How many seconds did the user take to complete the task?</td><td colspan="2"></td></tr>
<tr><td colspan="5">Expected path
Did the user follow the expected path?</td><td>Yes</td><td>No</td></tr>
<tr><td colspan="7">Expected path description
In the bottom bar, select the "Diary" option. Open details of a meal. Select the "Edit" option. Change the name of the meal. Click "Confirm"</td></tr>
<tr><td colspan="5">Errors and assistance
Did the user need help?</td><td>Yes</td><td>No</td></tr>
</table>

**The user is lost**<br>How many errors occurred and how did the user expect the application to behave?		
**Task #3**<br>**View the meal plan**		
**Execution time**<br>How many seconds did the user take to complete the task?		
**Expected path**<br>Did the user follow the expected path?	Yes	No
Expected path description<br>*In the drawer menu, select the "Meal Plan" option*		
**Errors and assistance**<br>Did the user need help?	Yes	No
**The user is lost**<br>How many errors occurred and how did the user expect the application to behave?		
**Task #4**<br>**Add a new entry to the physical activity diary**		
**Execution time**<br>How many seconds did the user take to complete the task?		
**Expected path**<br>Did the user follow the expected path?	Yes	No
Expected path description<br>*In the bottom bar, select the option "Exercise". Choose a day in the calendar. Fill in the required fields. Click on "Confirm"*		
**Errors and assistance**<br>Did the user need help?	Yes	No
**The user is lost**		

How many errors occurred and how did the user expect the application to behave?		
**Task #5**<br>**View notifications settings**		
**Execution time**<br>How many seconds did the user t take to complete the task?		
**Expected path**<br>Did the user follow the expected path?	Yes	No
Expected path description<br>*In the top bar, select the "Settings" option. Select the "Notifications" option in the menu.*		
**Errors and assistance**<br>Did the user need help?	Yes	No
**The user is lost**<br>How many errors occurred and how did the user expect the application to behave?		
**Task #6**<br>**View reports section**		
**Execution time**<br>How many seconds did the user take to complete the task?		
**Expected path**<br>Did the user follow the expected path?	Yes	No
Expected path description<br>*In the side menu select the "Reports" option*		
**Errors and assistance**<br>Did the user need help?	Yes	No
**The user is lost**<br>How many errors occurred and how did the user expect the application to behave?		

**Task #7**<br>**Logout from application**		
**Execution time**<br>How many seconds did the user take to complete the task?		
**Expected path**<br>Did the user follow the expected path?	Yes	No
Expected path description<br>*In the top bar select the "Logout" option – In the confirmation select the "Yes" option*		
**Errors and assistance**<br>Did the user need help?	Yes	No
**The user is lost**<br>How many errors occurred and how did the user expect the application to behave?		

Supplementary Table 3. Usability test script no. 3 for the backoffice "NutriClock"

Job / Study area:				
Sex:		Age:		
**Task #1**<br>**Log into the application with the administrator account indicated by the reseacher**				
**Execution time**<br>How many seconds did the user take to complete the task?				
**Expected path**<br>Did the user follow the expected path?			Yes	No
Expected path description<br>*On the login screen, fill in the fields and click on "Login"*				
**Errors and assistance**<br>Did the user need help?			Yes	No
**Task #2**<br>**Add an institution and then delete the added institution**				
**Execution time**<br>How many seconds did the user take to complete the task?				
**Expected path**<br>Did the user follow the expected path?			Yes	No
Expected path description<br>*In the sidebar, select the option "Institutions" – Click on "New Institution" – Fill in the name of the institution and click "Save" – See institution in the list-Click on "Delete" – Confirm deletion"*				
**Errors and assistance**<br>Did the user need help?			Yes	No
**Task #3**				

**In the user list, add a meal or a food item to a meal in the meal plan for today.**		
**Execution time**<br>How many seconds did the user take to complete the task?		
**Expected path**<br>Did the user follow the expected path?	Yes	No
Expected path description<br>*In the sidebar, select the option "Users" who have the NutriClock group option active. Choose the "Meal Plan" tab and follow the steps to add a meal or a food (it may vary depending on whether there is a meal registered for the same day or not)*		
**Errors and assistance**<br>Did the user need help?	Yes	No
**Task #4**<br>**Logout from application**		
**Execution time**<br>How many seconds did the user take to complete the task?		
**Expected path**<br>Did the user follow the expected path?	Yes	No
Expected path description<br>*In the top bar, select the "Logout" option*		
**Errors and assistance**<br>Did the user need help?	Yes	No
**Task #5**<br>**Log into the application with the professional account indicated by the researcher**		
**Execution time**<br>How many seconds did the user take to complete the task?		

**Expected path**<br>Did the user follow the expected path?	Yes	No
Expected path description<br>*On the login screen, fill in the fields and click on "Login"*		
**Errors and assistance**<br>Did the user need help?	Yes	No
**Task #6**<br>**Send a chat message to a user**		
**Execution time**<br>How many seconds did the user take to complete the task?		
**Expected path**<br>Did the user follow the expected path?	Yes	No
Expected path description<br>*In the top bar, select the "Chat" option. If there are no pending messages, select the option to view the message history. Select a patient and send a message*		
**Errors and assistance**<br>Did the user need help?	Yes	No

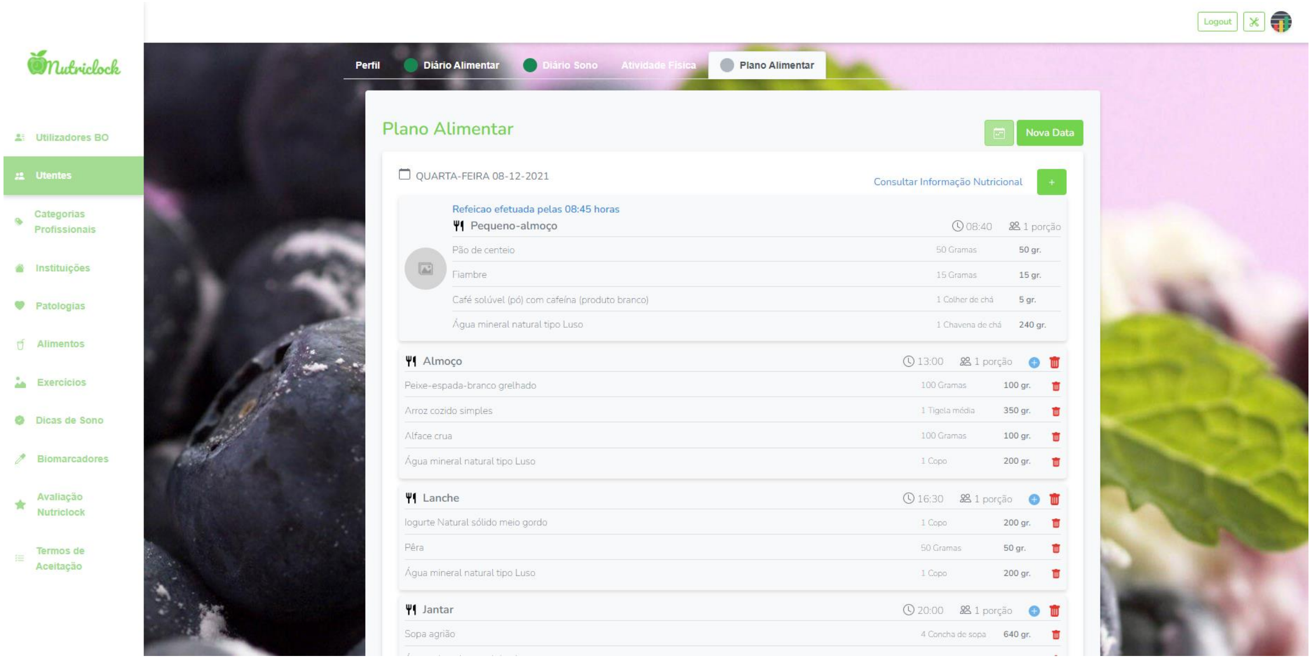

Supplementary Figure 1. Example of the NutriClock backoffice application's meal plan editing screen.

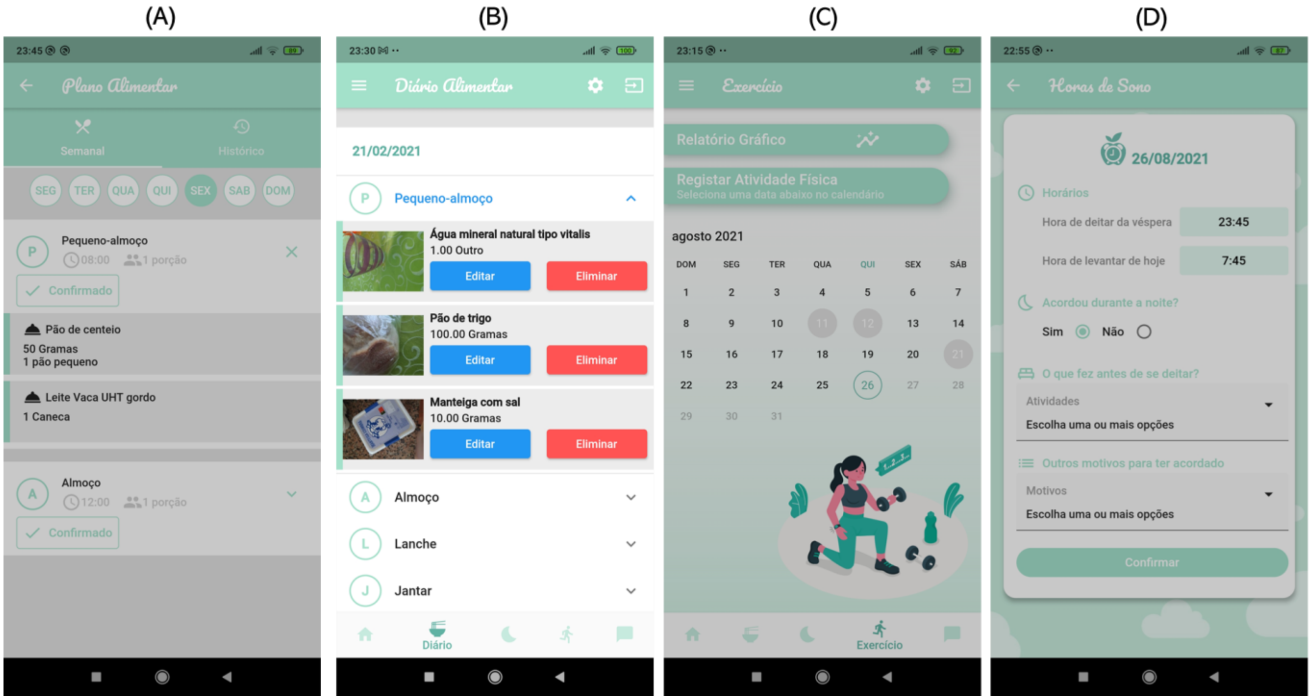

Supplementary Figure 2. Screenshots from the NutriClock mobile application. The screen in (A) is for viewing meal plans, the screen in (B) is for viewing the food diary, the screen in (C) is the physical activity diary, and the screen in (D) is for adding a new sleep diary entry.
